# Multimodal Insecticidal Activity of *α*-Humulene Against *Chilo suppressalis* via Endocrine Disruption and Transcriptional Suppression of Cuticular Proteins

**DOI:** 10.3390/ijms27188322

**Published:** 2026-09-18

**Authors:** Zhuo Peng, Wenhui Lu, Yue Meng, Junjie Lin, Xinyu Zhang, Zihan Guo, Rong Zhao, Xiaoying Li, Meican Liu, Qiaoying Zhou, Yuqian He, Rui Ji, Guirong Wang, Lei Yang, Yang Sun, Pengke Zhao

**Affiliations:** 1 Laboratory for Conservation and Use of Important Biological Resources of Anhui Province, Anhui Provincial Key Laboratory of Molecular Enzymology and Mechanism of Major Diseases, College of Life Sciences, Anhui Normal University, Wuhu 241002, China; pengzhuo0418@163.com (Z.P.); luwenhui1214@163.com (W.L.); 2321011721@ahnu.edu.cn (J.L.); 18171388351@163.com (X.Z.); gzh13355573529@163.com (Z.G.); 18756276526@163.com (R.Z.); 13987730492@163.com (X.L.); 18273395935@163.com (M.L.); 18048999716@163.com (Q.Z.); 18782770515@163.com (Y.H.); yanglei@jaas.ac.cn (L.Y.); 2 Agricultural Genomics Institute at Shenzhen, Chinese Academy of Agricultural Sciences, Shenzhen 518120, China; wangguirong@caas.cn; 3 Institute of Plant Protection, Chinese Academy of Agricultural Sciences, Beijing 100193, China; 4 School of Plant Protection, Anhui Agricultural University, Hefei 230036, China; mpriestj@163.com; 5 Institute of Plant Protection, Jiangsu Academy of Agricultural Sciences, Nanjing 210014, China; jirui@jaas.ac.cn; 6 Shaoxing Academy of Agricultural Sciences, Shaoxing 312000, China

**Keywords:** plant defense, *α*-humulene, *C. suppressalis*, transcriptomic

## Abstract

Striped rice stem borer (*Chilo suppressalis*, SSB) poses a substantial threat to global rice production, leading to considerable economic loss. In this study, we explored the insecticidal mechanism of *α*-humulene, a sesquiterpene derived from *Humulus lupulus*, against SSB larvae. Bioassays indicated that *α*-humulene markedly elevated larval mortality rates and extended developmental periods. Transcriptomic analysis revealed the upregulation of genes involved in juvenile hormone (JH) biosynthesis and the downregulation of cuticle protein genes. Molecular docking experiments demonstrated that *α*-humulene could form putative binding interactions with both cuticular proteins and key enzymes in the JH biosynthesis pathway, suggesting a potential interaction that may influence the structural stability of the cuticle and hormonal balance. Furthermore, JH and ecdysone titer assays confirmed hormonal imbalance in treated larvae. This study identified *α*-humulene as a novel multimodal insecticidal compound targeting two critical systems, namely endocrine disruption via dysregulated JH biosynthesis, and structural integrity impairment through cuticle protein suppression, presenting a sustainable approach for the eco-friendly management of rice pests.

## 1. Introduction

China’s ongoing stem borer outbreaks are primarily characterized by their extensive geographic spread, severe crop destruction, and substantial economic repercussions [1,2]. The epicenter of these infestations lies in the rice belt of the Yangtze River Valley, with the striped stem borer (SSB) *Chilo suppressalis* emerging as the dominant species, along with secondary infestations of the yellow stem borer (YSB) *Scirpophaga incertulas* [3,4]. SSB is a major lepidopteran pest that inflicts damage throughout the entire rice growth cycle, while also threatening secondary crops like *Zizania latifolia*, wheat, and corn. These extensive infestations severely compromise agricultural production, translating to an estimated annual economic loss of approximately 11.5 billion CNY [5].

Traditional reliance on synthetic insecticides such as chlorantraniliprole has led to significant resistance issues [6,7]. Field populations in China demonstrate remarkable resistance variability to chlorantraniliprole. Notably, the YY14 population from Zhejiang Province exhibited moderate resistance (a 77.6-fold resistance ratio), correlating with a novel G4910E mutation in the ryanodine receptor (RyR) gene identified through comparative sequencing [8]. Consequently, conventional pest management has become increasingly constrained by both rapidly evolving insecticide resistance and growing concerns over non-target ecological safety. For instance, chlorpyrifos exhibits non-target toxicity as its environmental persistence in aquatic systems may endanger various organisms through unintended exposure [9]. These challenges highlight the urgent need for eco-friendly alternatives that can bypass the resistance and mitigate ecological harm.

Plant-derived terpenoids have emerged as promising multi-target biopesticides due to their structural complexity and diverse modes of action [10,11]. Essential oils derived from *Humulus lupulus* demonstrate potential as novel control tools against mosquito vectors and stored-product pests owing to their richness in bioactive terpenes. *α*-Humulene, identified as a primary constituent, contributes significantly to this insecticidal activity through mechanisms requiring further elucidation [12,13]. Interestingly, previous studies have indicated a potential link between SSB infestation and *α*-humulene emission in rice: SSB feeding significantly induces the expression of the terpene synthase gene *TPS46* [14], and the overexpression of *TPS46* has been shown to result in a significant accumulation of *α*-humulene [15]. However, despite its established ecological role in plant–insect interactions, the mode of action of *α*-humulene in lepidopteran pests remains poorly understood; in particular, its potential capacity to disrupt vital developmental processes through multimodal mechanisms lacks comprehensive toxicological investigation.

In this study, we aimed to systematically investigate the direct insecticidal effects of *α*-humulene against SSB and elucidate its underlying molecular mechanisms. To address the urgent need for plant-derived compounds with alternative modes of action to conventional insecticides, and given the previously established role of rice-derived terpene volatiles in plant–insect interactions, we hypothesized that α-humulene functions as a multimodal bio-insecticide targeting essential physiological or developmental processes in SSB larvae. To test this hypothesis, we integrated toxicological bioassays with transcriptomic profiling, molecular docking, and endogenous hormone quantification. By exploring these multi-level interactions, this study seeks to advance our mechanistic understanding of terpenoid-based defense compounds. Ultimately, our findings provide a robust theoretical foundation for exploring the potential of α-humulene as a sustainable, eco-friendly candidate in integrated pest management (IPM) strategies.

## 2. Results

### 2.1. α-Humulene Decreased the Performance of SSB Feeding on Artificial Diets

**Figure 1:**
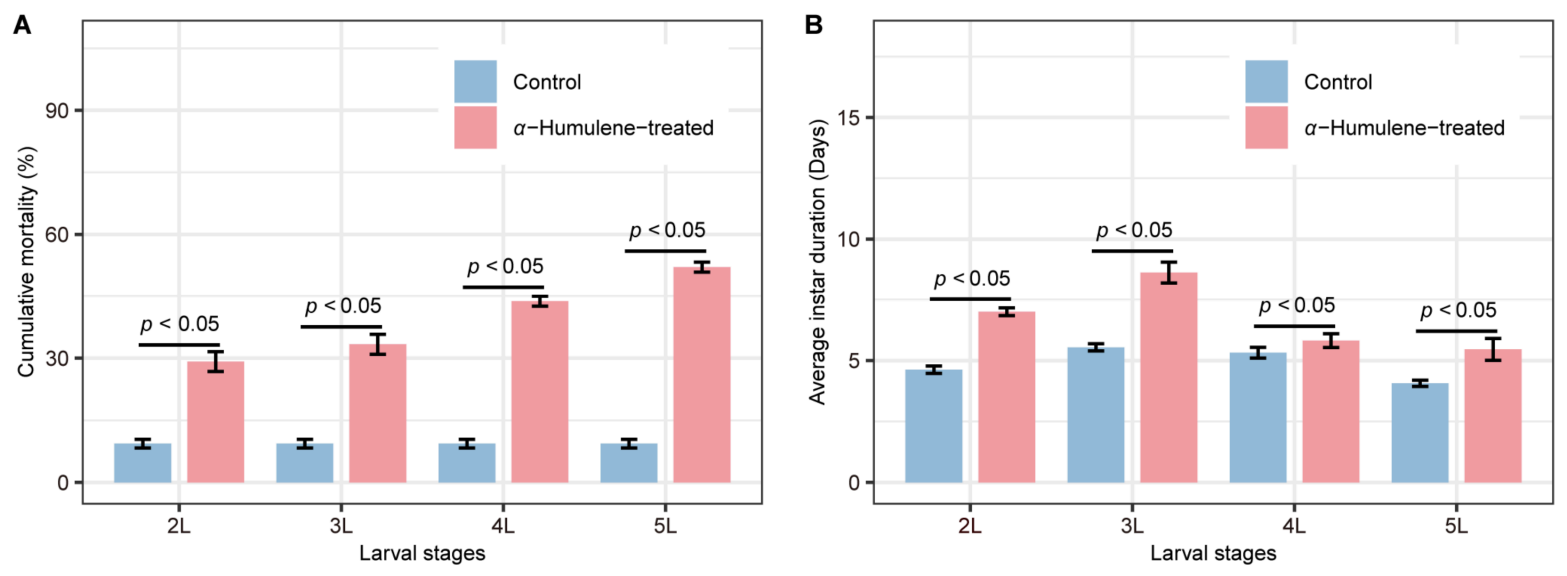
Effects of *α*-humulene exposure on larval mortality and developmental dynamics in the SSB. (**A**) Cumulative mortality of the SSB larvae reared on an artificial diet supplemented with *α*-humulene. (**B**) The duration of larval instars after *α*-humulene treatment. Data represent means ± SEM of five independent biological replicates.

The cumulative mortality rate of the second- to fifth-instar larvae were significantly higher than that of the control group (Figure 1A; *p* < 0.05). Notably, we observed a higher rate of mortality among second-instar larvae than among third- to fifth-instar larvae. Compared to the control, *α*-humulene treatment larvae showed developmental delays, with approximately half of the treated larvae failing to reach the pupal stage. Consequently, we determined the developmental time for each larval stage of the SSB. Our findings indicated that *α*-humulene significantly prolonged the duration of the second and third instar larval stages, increasing from 4.62 to 7.00 days and 5.54 to 8.63 days, respectively (Figure 1B; *p* < 0.05). In addition, larvae that ingested *α*-humulene exhibited abnormal pupation, resulting in malformed pupae. Collectively, these data suggest that *α*-humulene significantly inhibits larval development and/or induces abnormal larval phenotypes in SSB larvae.

### 2.2. α-Humulene Treatment Reshapes the SSB Larval Transcriptome

**Figure 2:**
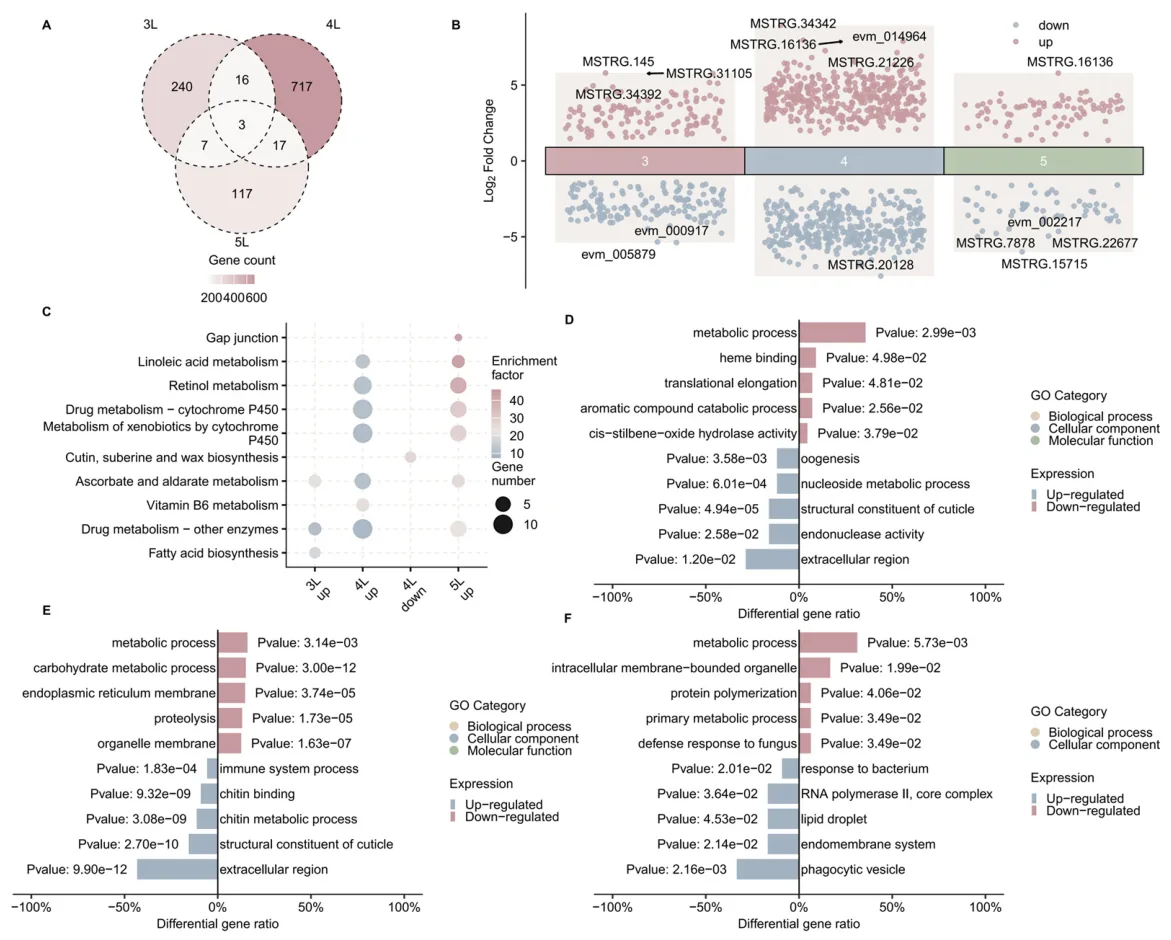
Genome-wide profiling of differentially expressed genes (DEGs) during larval development in SSB. (**A**) Venn diagram of overlapping DEGs between consecutive larval instars (L3–L5). (**B**) Volcano plot depicting DEGs between the third and fifth larval instars of SSB. Genes with |log_2_(fold change)| > 1 and FDR-adjusted *p*-value < 0.05 are highlighted in purple (upregulated) and blue (downregulated). The five most significant DEGs (labeled with gene IDs) were selected based on the combined fold-change. (**C**) Bubble plot of the top 10 KEGG pathway enrichment analysis for DEGs between the third and fifth larval instars of the SSB. Enriched pathways (*q*-value < 0.05) were analyzed separately for upregulated and downregulated DEGs using a hypergeometric test. Bubble size represents the number of DEGs enriched in each pathway, while color indicates the enrichment factor. Pathway annotations were retrieved from the KEGG database. (**D**) Symmetrical bar plots of Gene Ontology (GO) enrichment analysis of SSB larval responses to *α*-humulene. (**D**) 3rd, (**E**) 4th, and (**F**) 5th instars. For each instar, the top five significantly enriched GO terms (*p* < 0.05) were selected based on the differential gene ratio. Red bars represent treatment-upregulated terms and yellow bars represent treatment-downregulated terms. Text labels are colored by GO category: biological process (cyan), molecular function (lavender), and cellular component (sky blue). Raw *p*-values (uncorrected) are shown in grey.

To explore the mechanism by which *α*-humulene hinders SSB growth and development, we analyzed the transcriptome of *α*-humulene treatment larvae using RNA-seq. We next identified which SSB genes were differentially expressed in *α*-humulene treatment versus control larvae; a total of 266 DEGs were identified in third-instar larvae, 753 DEGs were identified in fourth-instar larvae, and 144 DEGs were identified in fifth-instar larvae (Figure 2A). Notably, there were only three genes shared between all three treatments, and between 10 and 20 genes shared between any two treatments (Figure 2A). Differentially expressed genes with the highest absolute log_2_FC were plotted using a volcano plot (Figure 2B). KEGG functional enrichment analysis showed that up-regulated DEGs were significantly enriched in “Pentose and glucuronate interconversions”, “Drug metabolism-other enzymes”, and “Ascorbate and aldarate metabolism pathways”; these pathways were observed in all three treatments (Figure 2C). Additionally, downregulated DEGs were only enriched in a few pathways, with “Amino sugar and nucleotide sugar metabolism” appearing in the 3rd instar larvae and Cutin, suberine, and wax biosynthesis pathways appearing in the 4th instar larvae (Figure S1; Figure 2C). Furthermore, we found that the insect hormone biosynthesis pathway, which is crucial for insect growth and development, was significantly enriched in the fourth-instar larvae (Figure S1). Next, we performed a GO enrichment analysis of DEGs (Figure 2D–F). The DEGs encompassed most of the major biological processes for the “metabolic processes”, which were the most enriched groups (Figure 2D). Compared to the control, the structural constituent of the cuticle was the most significantly enriched term among the downregulated DEGs in the 3rd and 4th instar larvae (Figure 2D,E). Chitin-related processes that are critical for insect growth, development, and metamorphosis were significantly inhibited (Figure 2E).

Given the critical role of insect hormones in regulating growth, development, and reproduction, we have conducted an in-depth analysis of the biosynthetic pathways involved in the insect hormone biosynthesis pathway (Figure S2). The heatmap illustrates the transcriptional signatures of the selected genes in SSB after *α*-humulene treatment. For JH, we found that all differential genes were upregulated (Figure S2), while some genes involved in ecdysone remained unchanged. The observed changes may be indicative of a hormone imbalance in SSB larvae following *α*-humulene treatment. Validation of the RNA sequencing data for six random genes by quantitative RT-qPCR supported the reliability of our transcriptome data (Figure S3).

### 2.3. α-Humulene Suppresses Cuticle-Related Gene Transcription and Interacts with Juvenile Hormone Biosynthesis-Associated Proteins

**Figure 3:**
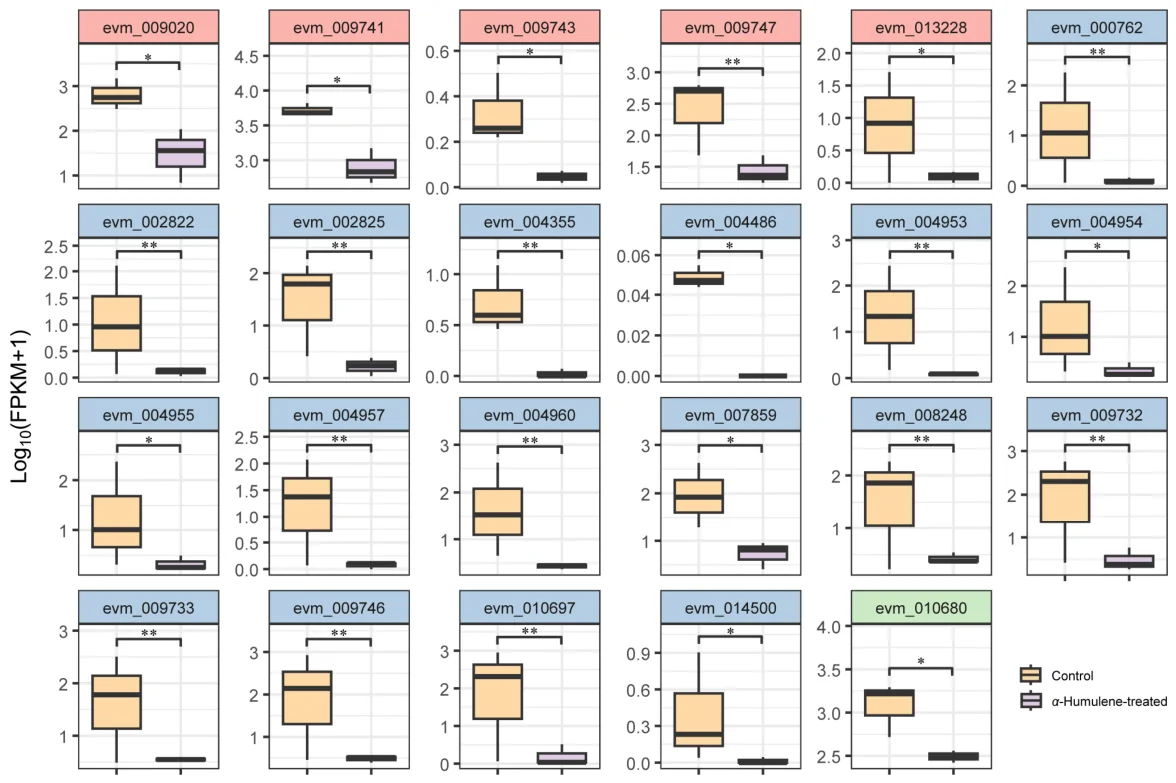
Systematic analysis of cuticle protein gene suppression in SSB larvae following *α*-humulene treatment. The annotation of all cuticle protein genes was based on sequence homology searches against the NR and UniProt databases, with quantitative analyses of gene expression performed using normalized FPKM values throughout this study. *, *p* < 0.05; **, *p* < 0.01.

Functional enrichment analysis was performed for the identified DEGs and revealed the enrichment of cuticle-associated GO terms and KEGG pathways (Figure 2C,D). We then searched the DEGs for cuticle-related genes and manually reviewed the genes. A total of 23 genes associated with the cuticle were identified according to current research, and all of them were downregulated. Cuticles are essential for insect growth and development. Our results strongly indicated that *α*-humulene may inhibit the transcriptional levels of insect cuticular proteins, thereby preventing SSB larval metamorphosis (Figure 3).

**Figure 4:**
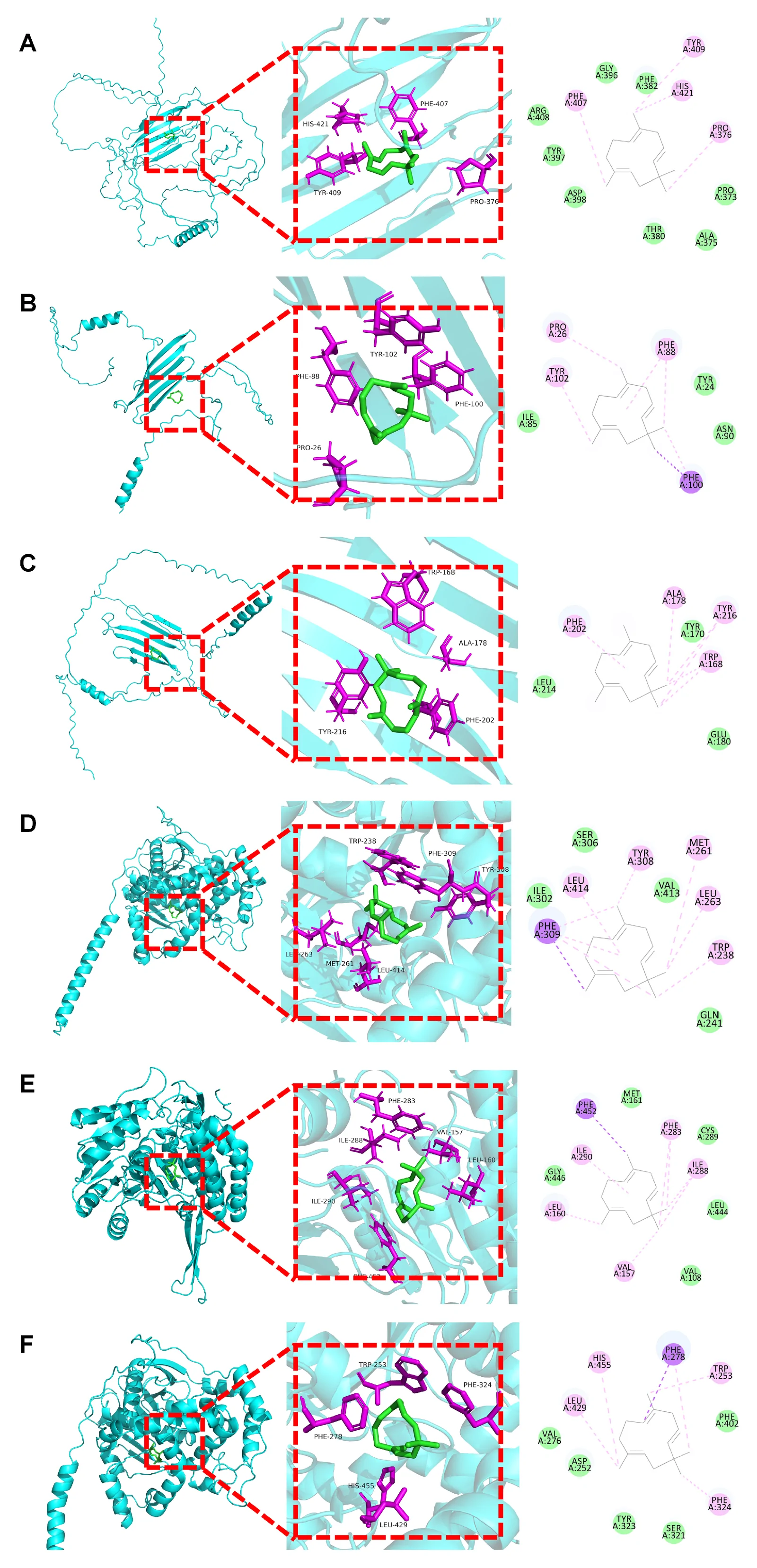
Molecular interaction analysis between *α*-humulene and the target protein. (**Left**) Binding conformation diagram. The protein is represented as a cyan ribbon diagram, while *α*-humulene is depicted as a green stick model. (**Middle**) Three-dimensional interaction map. The protein (cyan ribbon) and *α*-humulene (green stick) are shown, with hydrophobic interacting residues highlighted as pink spheres. (**Right**) Two-dimensional interaction diagram. Color coding: Green, van der Waals interactions; pink, hydrophobic interactions; purple, Pi-Sigma interactions. (**A**), evm_004355; (**B**), evm_013228; (**C**), evm_009747; (**D**), evm_011721; (**E**), evm_013436; (**F**), evm_010323.

To further elucidate the interaction between *α*-humulene and cuticular proteins in the SSB, molecular docking studies were systematically performed on all differentially expressed cuticular protein genes. Our analyses revealed that *α*-humulene exhibited moderate binding potential with selected cuticular proteins (Figure 4A–C). As exemplified by evm_004355 (binding affinity: −6.331 kcal/mol), the compound demonstrated complementary shape matching within the folded structural domain of the protein (Figure 4A). Molecular interaction analysis identified four stable hydrophobic interactions and multiple van der Waals forces between the ligand and target protein, though the absence of hydrogen bonding interactions was notable. Key amino acid residues mediating hydrophobic contacts included PHE407, TYR409, HIS421, and PRO376 (Figure 4A). Similar binding patterns were observed in other cuticular proteins (evm_013228 and evm_009747), suggesting a conserved interaction mechanism (Figure 4B,C).

Given the critical role of JH biosynthesis-associated proteins in insect development, parallel docking studies were conducted on these targets (Figure 4D–F). The representative protein evm_010323 demonstrated a binding affinity of −7.784 kcal/mol, indicative of moderate interaction strength (Figure 4F). Structural analysis revealed the compound’s occupation of an internal pocket domain with favorable shape complementarity. Six distinct hydrophobic interactions and extensive van der Waals contacts were identified, while hydrogen bonding remained absent. Critical residues involved in hydrophobic stabilization included LEU429, HIS455, PHE278, TRP253, and PHE324 (Figure 4F).

### 2.4. α-Humulene Treatment Disrupted the Balance Between JH and Ecdysone in the SSB Larvae

**Figure 5:**
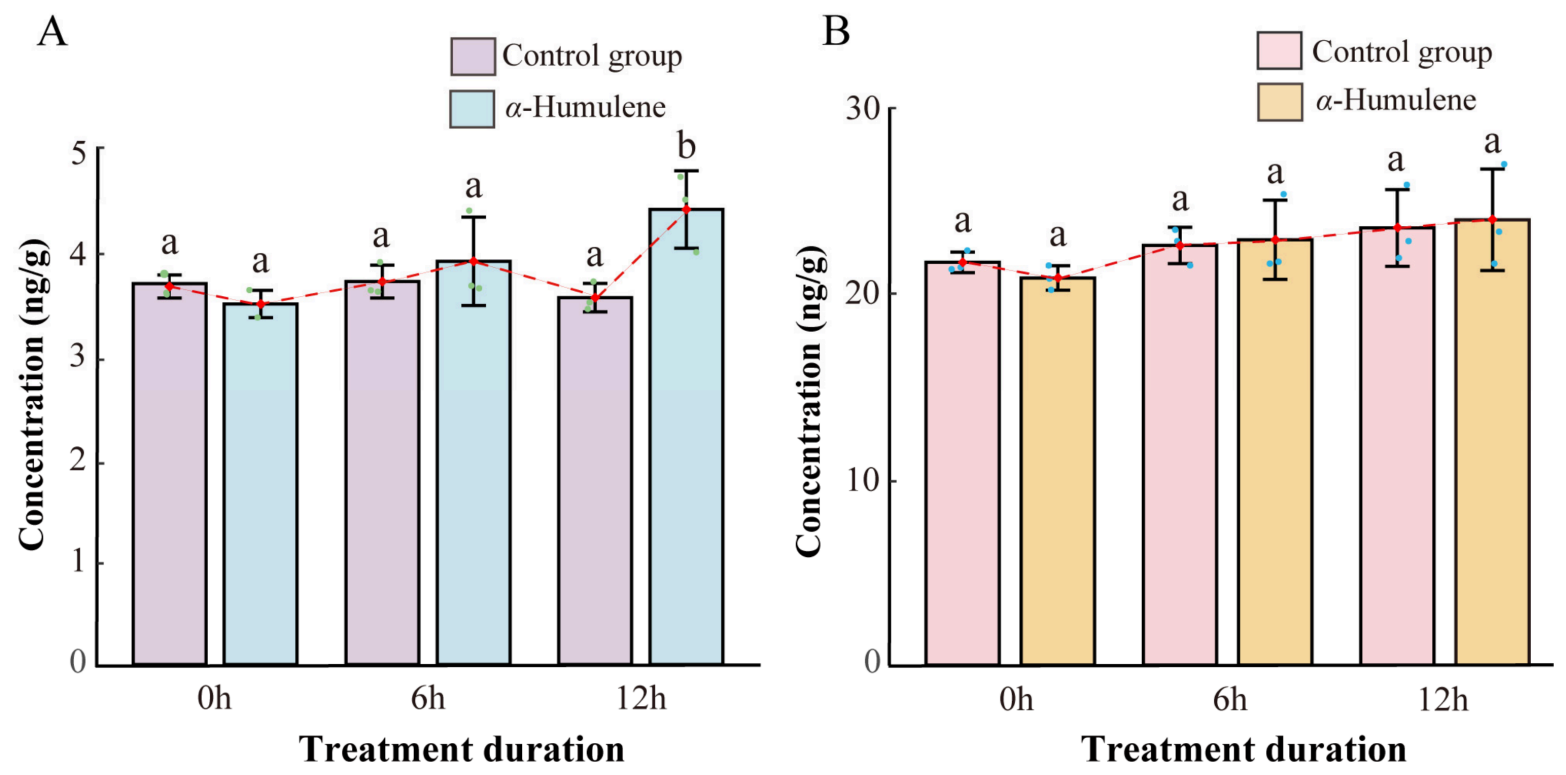
Effects of *α*-humulene exposure on JH and ecdysone titers in the SSB larvae. (**A**) Changes in JH titers in the SSB larvae with different *α*-humulene exposure durations. (**B**) Ecdysone titers in the SSB larvae with varying durations of *α*-humulene exposure. Data represent means ± SEM of five independent biological replicates. Lowercase letters indicate significant statistical differences (Tukey; *p* < 0.05).

Compared with the control group, no significant changes were observed in the JH titers in the *α*-humulene treatment 3rd instar larvae of SSB for 0 h and 6 h. However, a significant increase in JH titers in the larvae, from 3.719 ng/g to 4.311 ng/g, was presented in the data when the treatment duration with *α*-humulene was extended to 12 h. (Figure 5A, *p* < 0.05). Meanwhile, the ecdysone levels in 3rd instar larvae treated with *α*-humulene for 0 h, 6 h, and 12 h remained relatively stable (Figure 5B). These results, similar to those of transcriptomic analysis, suggested that *α*-humulene may disrupt the balance of endogenous hormones in SSB larvae by promoting the synthesis of JH. Moreover, under *α*-humulene stress, the changes in hormone titers in SSB larvae corresponded to the phenotypic changes observed in bioassays, suggesting that *α*-humulene likely inhibited larval growth and development by interfering with endocrine regulation, which may ultimately contribute to substantial larval mortality.

## 3. Discussion

Terpenoids, recognized as the largest class of plant secondary metabolites, exhibit remarkable biological diversity in plant defense mechanisms [16,17]. Building upon previous findings that *α*-humulene demonstrates insecticidal activity against mosquitoes and freshwater snails while serving as an eco-friendly repellent for stored-product pests [12,13], we investigated its potential against SSB, a devastating pest notorious for its stem-boring behavior that renders conventional chemical control ineffective. Our study indicates that *α*-humulene may exert potent insecticidal activity against the SSB through two distinct mechanisms: potential disruption of JH homeostasis (Figure 2C; Figure S2) and impairment of cuticular protein integrity (Figure 2D–F; Figure 3). This dual-target mode of action distinguishes *α*-humulene from conventional neurotoxic insecticides and aligns with the growing demand for multi-site biopesticides that may delay resistance development.

The upregulation of key enzymes in JH metabolism, including ALDH and JHEH, suggested that *α*-humulene interferes with JH titer regulation (Figure S2). Because JH-degrading enzymes serve as crucial regulators of endogenous JH titers, we interpret their transcriptional upregulation primarily as a compensatory feedback mechanism in which the larvae attempt to clear excess hormone and restore endocrine homeostasis under toxic stress. In *Aedes aegypti*, *AaALDH*3, an NAD^+^-dependent aldehyde dehydrogenase, critically regulates JH-dependent biological processes by maintaining a dynamic equilibrium between the potentially toxic farnesal and JH precursors [18]. In *Apolygus lucorum*, targeted RNAi knockdown of *AlJHEH* in 3rd instar nymphs significantly impaired molting progression with lethal cuticular malformations [19]. Our hormonal titer assays confirmed that *α*-humulene significantly alters the critical balance between JH and ecdysone by elevating JH titers while leaving absolute ecdysone levels relatively stable (Figure 5), providing physiological evidence for transcriptomic observations.

Cuticular proteins are essential structural components of insect exoskeletons that mediate the integration of biomechanical resilience with developmental plasticity through their dynamic expression patterns during sclerotization and molt cycles [20,21]. The CPR protein family, a crucial group of insect cuticular proteins, is categorized into three subgroups: RR-1, RR-2, and RR-3. RR-1 is potentially linked to soft cuticles, while RR-2 is associated with rigid cuticles [22,23]. In our study, the majority of suppressed cuticular proteins were identified as RR-1 and RR-2 members (Figure 3). Concurrently, systemic downregulation of 23 structural cuticular protein genes correlates with molecular docking evidence demonstrating humulene’s preferential binding to hydrophobic interfaces of cuticular proteins (Figure 4). The sesquiterpene’s macrocyclic architecture likely facilitates steric insertion into the chitin–protein matrices, disrupting their hierarchical assembly. This physicochemical interference may compromise cuticular integrity, manifesting as developmental aberrations distinct from those of neurotoxic insecticides (Figure 4).

The transcriptomic response to *α*-humulene exhibited a pronounced stage-specific pattern, characterized by a dramatic peak in differentially expressed genes (DEGs) during the fourth instar (753 DEGs), compared to the third (266 DEGs) and fifth (144 DEGs) instars, with minimal overlap across all three stages. This temporal dynamic suggests that the molecular impact of *α*-humulene is heavily dependent on the developmental stage, chronological age, and exposure duration. The substantial transcriptomic reprogramming observed in the fourth instar likely coincides with a critical physiological window. In lepidopterans, this stage involves profound endogenous hormonal shifts (such as fluctuations in JH and ecdysone titers) in preparation for the final larval molt and subsequent pupation [24,25]. Given our findings that *α*-humulene disrupts JH biosynthesis, the insect’s transcriptional machinery appears to be particularly vulnerable during this endocrine-sensitive phase. Furthermore, the escalation in DEGs from the third to the fourth instar may reflect cumulative toxicity resulting from prolonged dietary exposure. Conversely, the marked reduction in DEGs observed in the fifth instar could be attributed to enhanced developmental tolerance. Larvae that successfully reach the final instar may possess a higher baseline tolerance or have effectively mobilized detoxification systems, as supported by the enrichment of ‘drug metabolism’ pathways across treatments. Together, these stage-dependent variations highlight the highly dynamic and adaptive nature of the insect’s physiological response to *α*-humulene stress.

Our study elucidates the direct effects of *α*-humulene on SSB and preliminarily reveals its multifaceted modes of action against rice stem borers. Specifically, *α*-humulene exhibited significant lethal toxicity and developmental disruption by activating the JH pathway and suppressing chitin-related protein expression. Despite these advances, several limitations should be acknowledged. First, although molecular docking predicted stable binding between *α*-humulene and target proteins, biochemical validation (e.g., surface plasmon resonance or enzyme activity assays) is needed to confirm direct interactions. Second, laboratory bioassays were conducted under optimized conditions; field efficacy may vary due to environmental degradation, UV sensitivity, or plant matrix effects, and there is a lack of direct field trials to assess its practical application potential in agricultural ecosystems. Third, while the balance of JH and ecdysone titers was altered, the exact molecular link between *α*-humulene exposure and JH gene upregulation remains unclear. Transcriptomic time-series or promoter-reporter assays could address this. Finally, while this study focused on larval development, the long-term effects of *α*-humulene on adult emergence and reproduction remain unknown. Evaluating these later life stages will be an important next step for future research.

In conclusion, *α*-humulene is a highly promising compound for managing rice stem borers. It kills these pests by disrupting their natural hormone levels and damaging their outer shell structure. This makes it an excellent candidate for eco-friendly pest control. However, more work is needed before it can be used on farms. Future studies must test its performance in real fields, improve its formulation for better stability, and ensure it is safe for the environment. These practical steps will help determine how it can be best used in pest management.

## 4. Materials and Methods

### 4.1. Insect Rearing

The SSB populations analyzed in this study were originally collected from rice fields in Ruichang, Jiangxi Province, China. All insect colonies were reared under controlled laboratory conditions, maintained at 25 °C with 80% relative humidity, and exposed to a 14:10 h light–dark photoperiod cycle. Larvae were provisioned with an artificial diet formulated according to the methodology detailed in Han et al.’s protocol [26]. Upon emergence, adult insects were transferred to mating cages for reproduction. The adults were maintained under the same temperature and photoperiod (28 °C, 14:10 h light–dark photoperiod cycle), but the relative humidity was strictly maintained at 80% to facilitate optimal mating and oviposition. Additionally, adults were provisioned with a 10% honey solution on cotton to maintain longevity and fecundity.

### 4.2. α-Humulene Treatment

*α*-Humulene (CAS 6753-98-6) was commercially sourced from Sigma-Aldrich (St Louis, MO, USA). The treatment concentration of 0.8 g/kg was selected based on previous experimental settings [27]. The *α*-humulene-supplemented artificial diet was prepared by first dissolving *α*-humulene in an equal volume of absolute ethanol. This solution was then thoroughly blended into the artificial diet based on a mass ratio (*w*/*w*) to achieve a final concentration of 0.8 g/kg. A control diet was prepared simultaneously using an equivalent volume of ethanol alone. For each assay, we placed 24 newly hatched neonatal larvae into plastic rearing cups containing the respective diets. To prevent compound degradation and diet spoilage, the artificial diet was replaced daily (every 24 h) with freshly prepared ones. Larval mortality and developmental progression were scored daily (every 24 h) at a fixed time until pupation or death. Mortality was strictly defined as the lack of coordinated movement upon gentle stimulation with a fine brush. Pupal deformities were classified based on explicit morphological abnormalities, such as the failure to shed the final larval exuviae or severe distortion of pupal cases. To ensure reproducibility, control and treatment groups were maintained simultaneously under identical conditions. All bioassays were performed in four independent biological replicates. For subsequent transcriptomic and hormonal analyses, separate parallel cohorts were maintained under the same experimental setup. Once reaching the 3rd, 4th, and 5th instar stages, exactly 3 living larvae were pooled as one sample per biological replicate, with a total of three independent biological replicates systematically collected, snap-frozen in liquid nitrogen, and stored at −80 °C.

### 4.3. RNA Isolation and Library Preparation for Transcriptome Sequencing

SSB larvae were maintained on control diets or *α*-humulene-supplemented diets (0.8 g/kg) until they reached the third, fourth, and fifth instar stages, at which point whole-body specimens of the corresponding instars were collected and stored at −80 °C. Total RNA extraction was performed using TRIzol™ Reagent (Invitrogen, Carlsbad, CA, USA) according to the manufacturer’s protocols, with three biological replicates (six larvae per replicate) per treatment group. RNA integrity was verified by 1% agarose gel electrophoresis, followed by purity assessment using a NanoPhotometer spectrophotometer (IMPLEN, Westlake Village, CA, USA). RNA concentrations were quantified with a Qubit^®^ 2.0 Fluorometer (Thermo Fisher Scientific, Waltham, MA, USA) using the Qubit^®^ RNA assay kit. RNA integrity numbers (RINs) were determined using the RNA Nano 6000 assay on an Agilent 2100 Bioanalyzer system (Agilent Technologies, Santa Clara, CA, USA).

High-quality RNA samples (RIN > 8.0) were processed for library construction using the TruSeq™ RNA Library Prep Kit (Illumina, San Diego, CA, USA). Libraries were sequenced on an Illumina HiSeq platform with paired-end 150 bp reads. Adaptors were clipped and quality trimmed using FASTP 0.20.1 using default parameters (Q 30) [28]. HISAT2 (version 2.1.0) alignment software was used for reference genome mapping [29]. Transcript quantification was performed using StringTie with default parameters [30], followed by the calculation of FPKM values normalized by gene length and mapped read counts.

### 4.4. Differential Expression and Enrichment Analyses

Differential gene expression analysis was conducted using the edgeR package (v3.4.2) within the Bioconductor framework, employing negative binomial distribution models for count-based statistical comparisons [31]. Raw read counts were directly analyzed using the software’s integrated normalization algorithms to maintain the analytical precision. Statistical significance thresholds were set at false discovery rate (FDR)-adjusted *p*-values < 0.05, using the Benjamini–Hochberg multiple testing correction. Gene expression levels were quantified as fragments per kilobase of transcript per million mapped reads (FPKM). Although FPKM values were used for reporting gene abundance, differential expression analysis was performed using edgeR based on raw read counts.

The KEGG database (https://www.genome.jp/kegg/, accessed on 15 June 2024) was used to perform the enrichment analysis of pathways. Functional annotation clustering and Gene Ontology (GO) term enrichment were performed using the ClusterGVis package (https://github.com/junjunlab/ClusterGVis, accessed on 15 June 2024). All computational workflows and visualizations were developed using the R statistical environment (v4.4.2; https://www.r-project.org, accessed on 25 July 2026).

### 4.5. Quantitative RT-qPCR Analysis

Six randomly selected candidate genes were quantitatively validated to confirm the reliability of transcriptomic data. RNA specimens from the original sequencing experiment were reverse-transcribed into cDNA using the PrimeScript™ RT Reagent Kit (Takara Bio, Dalian, China) with three biological replicates per experimental group. Quantitative PCR amplification was performed using TB Green^®^ Premix Ex Taq™ (Takara) on a LightCycler^®^ 480 II system (Roche Diagnostics, Basel, Switzerland).

Gene-specific primers were designed using Primer Premier 6.0 (Premier Biosoft, CA, USA), with *EF1α* serving as the endogenous reference control [32]. Relative quantification was performed via the 2^−ΔCt^ method using instrument-integrated software. Primer sequences are cataloged in Supplementary Table S1. All reactions followed the MIQE guidelines for qPCR experimental reporting.

### 4.6. Determination of JH and Ecdysone Titers

Two separate ELISA kits (MLBIO Biotechnology, Shanghai, China) were used to determine JH and ecdysone levels in 3rd instar larvae of SSB via the double-antibody sandwich method, as this developmental stage exhibits the most pronounced phenotypic response. Three independent biological replicates were performed, each consisting of six larvae pooled for tissue homogenization. The collected larvae were washed, weighed, and homogenized on ice in pre-cooled homogenization buffer (0.01 M phosphate-buffered saline [PBS], pH 7.4) at a precise tissue-to-solution ratio of 1:9 (*w*/*v*; e.g., 0.1 g of tissue per 0.9 mL of PBS). The resulting homogenates were centrifuged at 5000× *g* for 10 min at 4 °C. The clear supernatants were collected and used directly without further dilution for the assays. According to the manufacturer’s specifications, the analytical sensitivity (minimum detectable dose) of the assays was <1.0 pg/mL for JH and <1.0 μg/mL for ecdysone. The dynamic detection ranges spanned from 6.25 to 200 pg/mL for JH and from 6.25 to 200 μg/mL for ecdysone. Insect hormone samples were added to the 96 microwells, where they combined with horseradish peroxidase (HRP)-labeled detection antibodies to form antibody–antigen–enzyme-labeled antibody complexes. After thorough washing, 3,3′,5,5′-tetramethylbenzidine (TMB) was added as a colorimetric substrate. Absorbance (optical density) was measured at 450 nm using a microplate reader, and hormone concentrations were calculated using standard curves.

### 4.7. Molecular Docking Analysis

The 3D structures of the target proteins (receptors) and the small molecule *α*-humulene (ligand) were initially formatted in PDB and SDF formats, respectively. Prior to docking, both the receptors and the ligand were preprocessed and converted into the AutoDock (version 4)-compatible PDBQT format using BatchVinaGUI software (version 2.2.0) [33]. For the target proteins, preprocessing steps included the removal of crystallization water molecules and non-protein structures, the retention of single conformations for multi-conformational residues, the deletion of lone electron pairs, the addition of all polar and non-polar hydrogen atoms, the reassignment of atomic types based on the AutoDock force field, and the calculation of Gasteiger charges. Because the specific ligand-binding pockets for these target proteins remain largely uncharacterized, a blind, semi-flexible docking strategy was employed using the AutoDock Vina 1.2.5 algorithm to allow an unbiased global search [34,35]. During this process, the protein structures were maintained rigidly while the ligand was permitted rotational flexibility along its rotatable bonds. The docking grid box was configured to encompass the entire protein structure to facilitate a comprehensive search for potential binding pockets across the whole protein surface. The optimal binding conformations were identified and ranked based on their lowest binding affinity scores (expressed in kcal/mol). Finally, the 3D binding conformations and 2D ligand–receptor interaction diagrams were generated and analyzed using PyMOL (version 3.1.0; Schrödinger, LLC, New York, NY, USA) and BIOVIA Discovery Studio Visualizer 2024 (Dassault Systèmes, Vélizy-Villacoublay, France).

### 4.8. Statistical Analysis

All statistical computations were performed using SPSS Statistics (version 24.0; IBM Corp., Armonk, NY, USA). Prior to analysis, data were evaluated for normality and homogeneity of variance using the Shapiro–Wilk test. Developmental durations within specific instar stages were analyzed using the non-parametric Mann–Whitney *U* test. Relative gene expression levels from RT-qPCR validation were analyzed using Student’s *t*-test. For other parameters, including cumulative mortality rates and hormone (JH and ecdysone) titers, statistical differences among treatments were analyzed using a one-way analysis of variance (ANOVA), followed by Tukey’s honestly significant difference (HSD) post hoc test. All quantitative data are presented as the mean ± standard error of the mean (SEM). Statistical significance was established at *p* < 0.05 and *p* < 0.01 (Note: The statistical models and criteria used for genome-wide differential gene expression from the RNA-seq data are detailed in Section 4.4).

## Data Availability

All raw sequencing data are available at the NCBI SRA under BioProject PRJNA1058995 and PRJNA1059269. Partial sequencing data are available at the NCBI GEO (http://www.ncbi.nlm.nih.gov/geo) under the accession number GSE179532.

## Supplementary Materials

The following supporting information can be downloaded at https://www.mdpi.com/article/10.3390/ijms27188322/s1.
