# Supplementary material for: Multimodal Insecticidal Activity of α-Humulene Against Chilo suppressalis via Endocrine Disruption and Transcriptional Suppression of Cuticular Proteins
Source: Int J Mol Sci. 2026 Sep 18;27(18):8322. doi: 10.3390/ijms27188322 (PMC13607974; doi:10.3390/ijms27188322)
Supplement: Supplementary file 1 [file ijms-27-08322-s001.zip › ijms-4498654-supplementary.pdf]

**Supporting Information**

Title: Multimodal Insecticidal Activity of α-Humulene Against *Chilo suppressalis* via Endocrine Disruption and Transcriptional Suppression of Cuticular Proteins

Table S1. The primers used in the qPCR analysis.

| Gene | Forward primer (5'-3') | Reverse primer (5'-3') |
|---|---|---|
| evm_008789 | AAGTGCCTTATGCTGTCAA | GTGGAAGTCATCAGTCTCAA |
| evm_008995 | TTGTGCTCAACGATGGAA | CACCGTAGCCTTCAATGT |
| evm_009157 | GCGATTATACAAGGAACTACC | TGTGTCTGCGTCAATAGC |
| evm_012791 | GAGCGATGTTACCGATGAA | CAGTGTCCGTCCAGTAGA |
| evm_013436 | ATGACCATCGCTAAGGAAG | GACCGTAGTTCGTGTTGT |
| evm_013945 | AGACAGACAGTGATAGTGATAC | GAGGTGATGGTGCGTTAG |

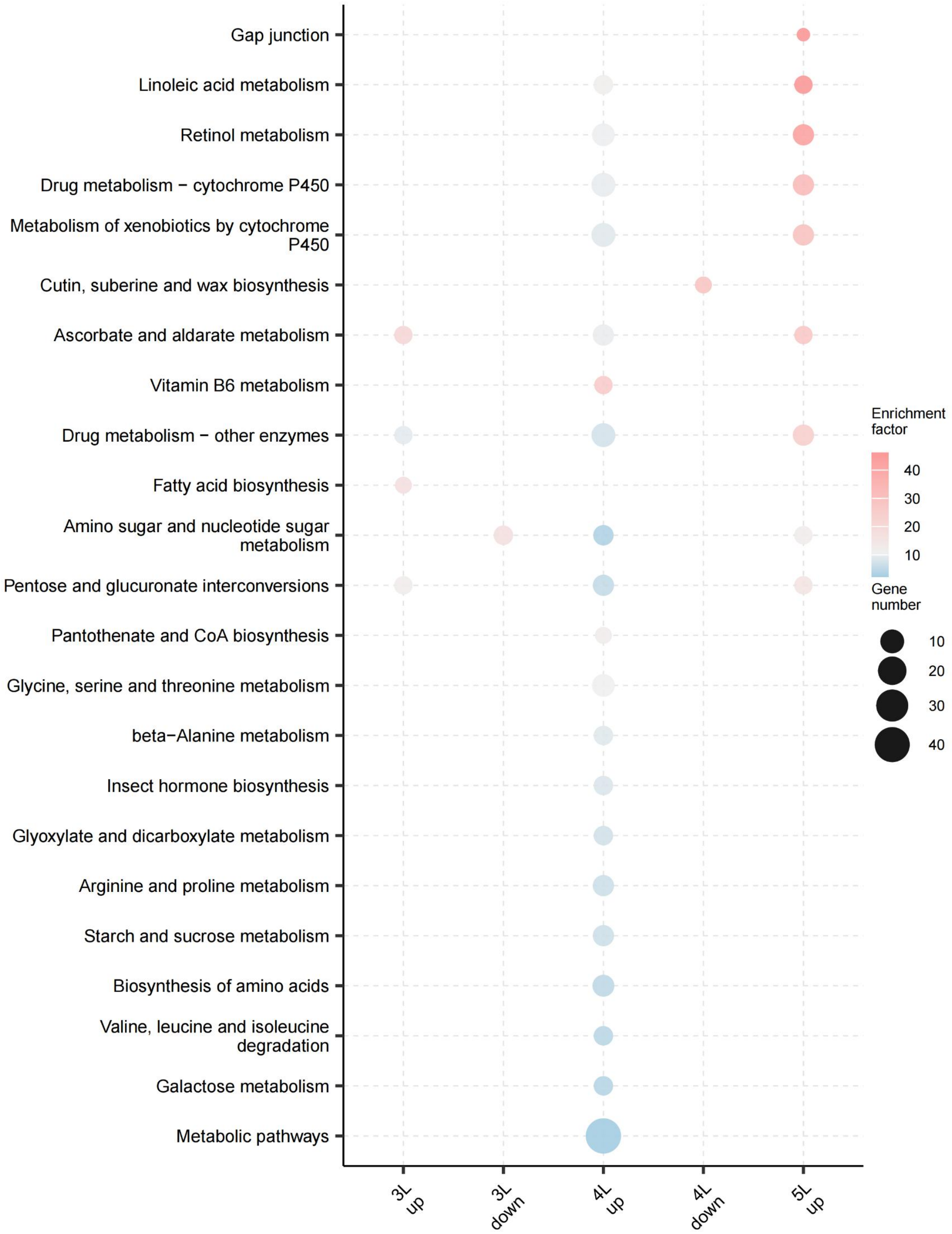


**Figure S1** Bubble plot of KEGG pathway enrichment analysis for DEGs between the third and fifth larval instars of the SSB. Enriched pathways (q-value < 0.05) were analyzed separately for upregulated (red bubbles) and downregulated (blue bubbles) DEGs using a hypergeometric test. Bubble size represents the enrichment factor, and the color intensity scales from red (most significant, *q*-value = 0.01) to blue (threshold significance, *q*-value = 0.05). Pathway annotations were retrieved from the KEGG database.

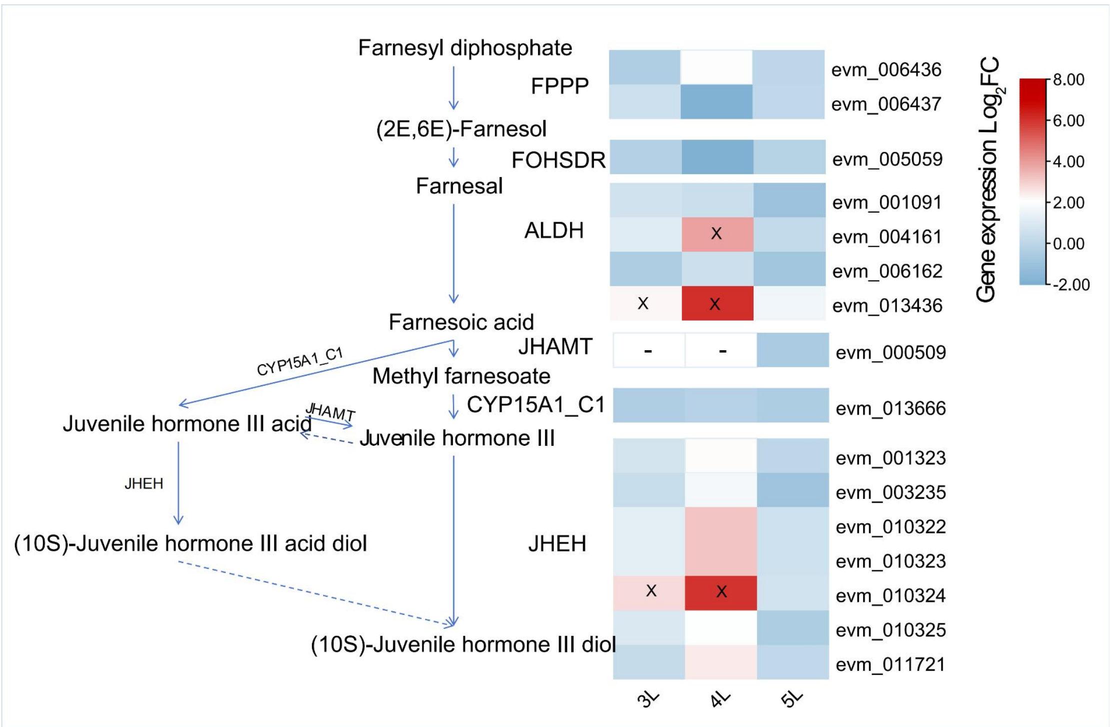


**Figure S2** Heatmap analysis of juvenile hormone biosynthesis pathway enzyme gene expression in SSB larvae after α-humulene treatment. Red, upregulated gene expression; blue, downregulated gene expression. Genes marked with "×" in the heatmap squares are significantly upregulated (fold change > 2, padj < 0.05). FPPP, farnesyl diphosphate phosphatase; FOHSDR, NADP+-dependent farnesol dehydrogenase; ALDH, aldehyde dehydrogenase (NAD+); JHAMT, farsoic acid methyltransferase; CYP15A1_C1, methyl farnesoate epoxidase / farnesoate epoxidase; JHEH, juvenile hormone epoxide hydrolase.

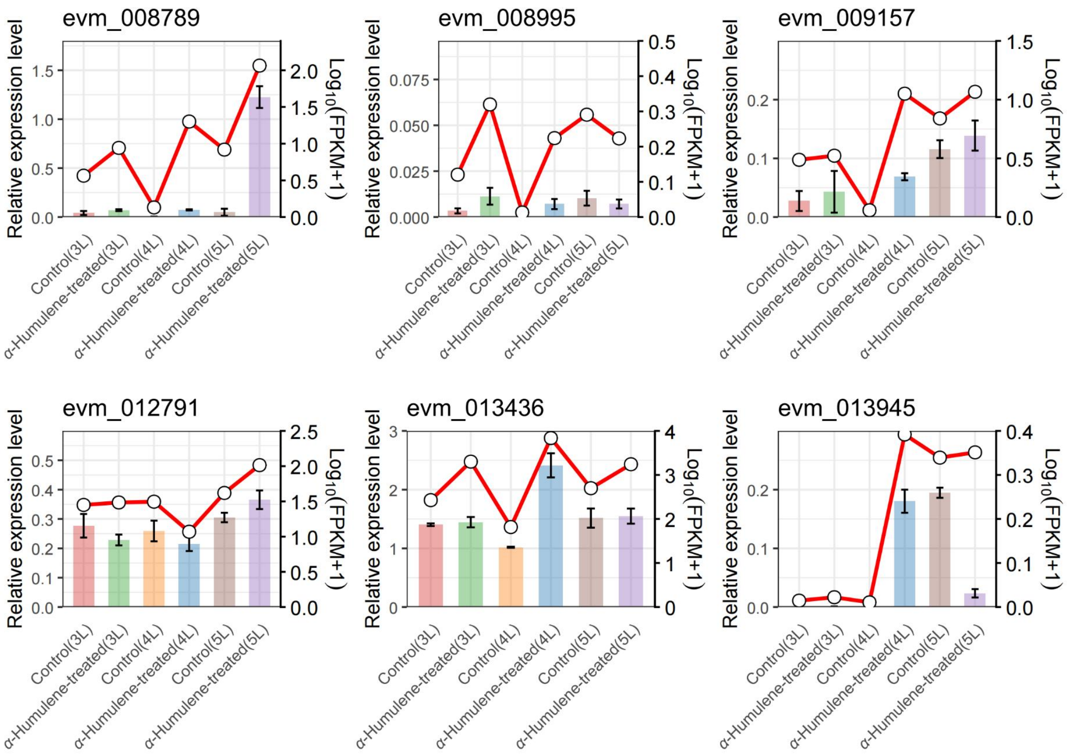


**Figure S3** RT-qPCR validation of RNA-Seq genes. RT-qPCR results (column chart) were compared with RNA-Seq data (red lines). The relative expression levels revealed in the RT-qPCR analysis are presented along the y-axis to the left, while the normalized expression levels (FPKM) of RNA-Seq genes are presented along the y-axis to the right.
